# Neural correlates of spatial orientation in a territorial frog *Allobates femoralis*

**DOI:** 10.1007/s00359-026-01820-6

**Published:** 2026-07-21

**Authors:** Andrius Pašukonis, Eva K. Fischer, Lauren A. O‘Connell

**Affiliations:** 1 Institute of Biosciences, Life Sciences Center, Vilnius University, Vilnius, Lithuania; 2 Department of Interdisciplinary Life Sciences, Konrad Lorenz Institute of Ethology, University of Veterinary Medicine Vienna, Vienna, Austria; 3 Department of Neurobiology, Physiology and Behavior, University of California Davis, Davis, CA USA; 4 Department of Biology, Stanford University, Stanford, CA USA; 5 Wu Tsai Institute for Neuroscience, Stanford University, Stanford, CA USA

**Keywords:** Navigation, Spatial cognition, Medial pallium, Amphibian

## Abstract

The brain regions supporting spatial navigation are well studied in mammals, but their function in the spatial behavior of other vertebrates is poorly understood, especially in field conditions. Here, we measured the behavior and neural correlates of the initial stages of navigation in a territorial rainforest frog, *Allobates femoralis,* in its natural environment. Frogs were released in familiar, unfamiliar, or home areas. Only frogs released in a familiar area, but outside their home territory, showed significant orientation towards home and spent more time on elevated structures, presumably orienting in space. In contrast, frogs released back in their home territory tended to move little, whereas those released in an unfamiliar area tended to move more, possibly exploring unfamiliar sites to find familiar landmarks. Contrary to our prediction, the amphibian homolog of the hippocampus (medial pallium) did not show a selective response to the navigational task. When accounting for behavioral differences, most measured pallial and subpallial regions showed increased neural activity during frog orientation home from a familiar environment. Interestingly, despite the behavioral differences, there were few differences in brain activity between the frogs released directly back home and those released in an unfamiliar area. Overall, we find that wild poison frogs respond selectively to environmental familiarity and that most measured brain regions have more translationally-active neurons when challenged to navigate a familiar setting. Our findings support the hypothesis that, in amphibians, brain regions homologous to mammalian centers of spatial processing exhibit more task-general responses.

**Supplementary Information:**

The online version contains supplementary material available at https://doi.org/10.1007/s00359-026-01820-6.

## Introduction

Bridging the gap between descriptions of behavior in the field and controlled mechanism-oriented experiments in the lab is a major and persistent challenge in biology. Peter Narins has been a pioneer in closing this gap by bringing mechanistic questions and rigorous experiments to wild places and animals, most notably rainforest amphibians. Among several examples, Peter Narins, working with Walter Hödl, established a small and obscure Amazonian rainforest frog, *Allobates femoralis* (previously *Epipedobates femoralis*), as a model species for bioacoustical field research. Their pioneering work explored how visual and auditory integration shapes recognition, aggression, and orientation in territorial male frogs (Hödl et al. 2004; Narins et al. 2003, 2005). Twenty years later, *A. femoralis* has been used as a model species for field research in biogeography, parental behavior, movement ecology, and navigation. Inspired by and building on the foundational experimental field studies of Narins and Hödl in this system, we here examine the neural correlates of spatial behavior in *A. femoralis*.

In vertebrates, the cognitive and neural mechanisms of spatial behavior have been studied primarily in mammals and, to a lesser extent, in birds (Jacobs and Schenk 2003; Herold et al. 2015; Bingman and Muzio 2017). In mammals, the hippocampal formation and the surrounding entorhinal cortex are the main neural substrates for encoding of space by several direction- and position-tuned classes of neurons (O’Keefe 1976; O’Keefe and Nadel 1979; Hafting et al. 2005; Moser et al. 2008; Geva-Sagiv et al. 2015). In addition, exploration and spatial orientation by route-following and path-integration rely on coordinated activity with subpallial structures, particularly the striatum and the septum (Packard and McGaugh 1996; McNaughton et al. 2006; Chersi and Burgess 2015; Hinman et al. 2018). Developmentally and functionally homologous mechanisms that support spatial learning and navigation have also been demonstrated in birds (Bingman et al. 2005; Jacobs and Menzel 2014; Herold et al. 2015). Overall, the vertebrate forebrain is structurally highly diverse, but evidence is accumulating that some areas responsible for spatial coding, such as the hippocampal formation and its non-mammalian homologs, have been functionally—if not structurally—conserved across vertebrates (Salas et al. 2003; Broglio et al. 2015; Bingman and Muzio 2017). Indeed, several lesion and immunohistochemical studies have shown that corresponding pallial areas play an important role in spatial memory in teleost fish (Salas et al. 1996; Rodríguez et al. 2021), amphibians (Sotelo et al. 2022, 2024), and non-avian reptiles (Rodríguez et al. 2002; López et al. 2003). However, the neural basis of spatial behavior in these vertebrate groups remains much less studied, especially under natural conditions, hindering our understanding of how spatio-cognitive abilities have evolved.

Amphibians are the most ancient terrestrial tetrapod group and therefore, a key taxon to understand the evolution of vertebrate spatial cognition. They also offer a tractable study system for navigation behavior and its underlying neural mechanisms. Robust navigational ability and homing behavior to aquatic breeding sites and terrestrial home ranges is widespread and well-studied in amphibians (Ferguson 1971; Sinsch 1990, 2006; Shaykevich et al. 2022; Pašukonis et al. 2022). The neural basis of amphibian spatial behavior has been almost exclusively studied in a terrestrial toad, *Rhinella arenarum* (Sotelo et al. 2017, 2020, 2022). These neuroethological experiments (reviewed in Sotelo et al. 2024) along with connectivity studies (Bruce and Neary 1995; Westhoff and Roth 2002; González et al. 2020) suggest that pallial regions including the medial pallium (Mp) (putative homolog of the mammalian hippocampus) are associated with a variety of behaviors that require sensorimotor integration and associative learning (Dicke and Roth 2007; Striedter and Northcutt 2019). Sotelo et al. (2024) also highlight the roles of the lateral and dorsal pallia, striatum, and medial septum as mammalian homologs potentially important in amphibian spatial navigation, but the studies incorporating these brain regions are even more scarce (Wenz and Himstedt 1990; Ruhl et al. 2016; Sotelo et al. 2017; Shaykevich et al. 2025). Only one recent field study, also in a toad (*Rhinella marina*), has implicated both the lateral and medial pallia in amphibian spatial orientation (Shaykevich et al. 2025). Therefore, as in most vertebrates, it is unknown whether navigation in the wild implicates a similar neural network as spatial tasks in controlled, small-scale studies.

Neotropical poison frogs (Dendrobatoidea) are a group of small terrestrial frogs with complex spatial behavior that have increasingly gained attention in studies of amphibian spatial cognition in the field (Stynoski 2009; Pašukonis et al. 2016, 2022) and in the lab (Liu et al. 2016, 2019). Most poison frogs defend small territories on the forest floor and transport their tadpoles on their backs from terrestrial egg-laying sites to scattered aquatic nurseries in the surrounding area (Summers and Tumulty 2014; Stynoski et al. 2015). Spatial learning plays a crucial role in their ability to find forest pools for tadpole deposition and the way back home (Pašukonis et al. 2014, 2016, 2019). When experimentally translocated from home territories, poison frogs show a robust homeward orientation and direct map-like navigation back home (Pašukonis et al. 2014, 2018). The details of homing behavior have been particularly well-studied in the brilliant-thighed poison frogs (*Allobates femoralis*)*.* When translocated up to 200 m, territorial *A. femoralis* males first show a stationary phase, the duration of which increases with translocation distance, suggesting that this period is key for successful orientation (Pašukonis et al. 2014, 2022). Following this stationary period, most frogs return in a direct path. However, when frogs are released in unfamiliar areas, they show no homeward orientation and instead move in an exploratory loop-like fashion (Pašukonis et al. 2014).

We used this robust homing response of male *A. femoralis* to investigate the neural correlates of the initial stage of spatial orientation. We collected the brains of frogs during this stationary orientation phase and used immunohistochemistry to evaluate the neural correlates of three different experimental release conditions: capture and release in a familiar environment, an unfamiliar environment or in their home territory. We predicted that the medial and dorsal pallia (Mp and Dp) would show an increased neural activity in frogs released in a familiar area, but not when released back home or in an unfamiliar site. In contrast, we expected that the striatum (Str) and septum (Sep) would show increased neural activity in frogs released in an unfamiliar environment, because these regions are typically associated with exploration, route-following, and path integration (Packard and McGaugh 1996; Chersi and Burgess 2015; Mayer et al. 2016; Ruhl et al. 2016; Hinman et al. 2018). The homology and function of the lateral pallium (Lp) in amphibians is poorly understood, but this region has strong afferent and efferent connections with the olfactory bulb (Bruce and Neary 1995; Westhoff and Roth 2002; González et al. 2020), and could be involved in a variety of behaviors, including territorial recognition and navigation. Finally, because the preoptic area (Poa) of the hypothalamus is involved in social behaviors, such as territoriality and parental care (Demski and Knigge 1971; Numan and Insel 2003; Ruscio and Adkins-Regan 2004; Fischer et al. 2019), we expected increased neural activation of the Poa in frogs released back in their territory.

## Materials and methods

### Study site and species

The experiments were carried out from January 27 to February 24 2017 in the area surrounding the field camp Saut Pararé (4°02*′* N, 52°41*′* W) at the Nouragues Ecological Research Station in the Nature Reserve Les Nouragues, French Guiana. The study area is largely composed of primary lowland rainforest bordering the Arataï river. *Allobates femoralis* (Boulenger 1884) is a small diurnal leaf-litter frog distributed and abundant throughout the Amazonian basin and the Guiana Shield (Amézquita et al. 2009). The species is diurnal and active from sunrise (~ 06:40 local time). Territorial males defend and vocally advertise territories on small patches of the forest floor with average distances between neighbors at our study site of ~ 9 m (Rodríguez et al. 2020). Mating takes place inside the male’s territory and tadpoles are transported primarily by males to a variety of small pools up to 200 m away from the territory litter (Beck et al. 2017; Roithmair 1992; Ringler et al. 2013).

### Frog selection and tagging

A total of 42 territorial males were captured and tagged. Frogs were found and captured during their peak calling activity within two hours before sunset. We identified territorial males by their calling and by exposing them to a playback of a simulated territorial intruder. Territorial males react to territorial intruders by a conspicuous phonotactic approach of the speaker (Amézquita et al. 2005). Frogs were captured and placed in an airtight opaque plastic 3.5 L barrel with damp leaf litter collected in an area without *A. femoralis*. Plastic barrels were cleaned between uses. Frogs were kept within plastic bags for up to 30 min before tagging and handled for less than five minutes during tagging. We used a previously established tracking technique consisting of a hand-held harmonic direction finder (R8 and R9, Recco AB, Lindigö, Sweden) and a custom-made passive transponder attached to the frogs with a silicone waistband (Pašukonis et al. 2014; Beck et al. 2017). The tag weighed ~ 0.1 g and accounted for ~ 5% of adult *A. femoralis* weight. Tagged frogs have been previously observed to perform all natural behaviors and navigate from long distances (Pašukonis et al. 2022). The capture site (i.e. home territory) was marked with a flagging tape and recorded with a GPS device MobileMapper 10 (SpectraPrecision, Westminster, CO, USA), using point averaging (30 GPS fix average) to increase GPS accuracy. Frogs were then held within the plastic barrel holding containers overnight at a forest edge of the field station with no territorial males calling in the vicinity.

### Experimental conditions and behavior

The next morning after capture, 50 to 90 min after sunrise, tagged frogs were released from the holding containers under one of the three experimental conditions or kept inside the container as a control group. We define the four groups as follows:

1. **Home (n = 10):** Frogs released back in their home territory within three meters from the capture site. These frogs are released in a familiar environment but do not need to navigate.
2. **Familiar (n = 9):** Frogs released ~ 50 m from the capture site. Translocation of territorial males to a familiar area typically induces homeward orientation and homing. Territorial males move up to ~ 200 m from home, and most males translocated 50 m return directly home within 1 to 38 h (10 h on average) (Pašukonis et al. 2014, 2022)
3. **Unfamiliar (n = 16):** Frogs released in an unfamiliar area on a 5-hectar river island approximately 200 to 600 m from respective capture sites. Frogs do not naturally cross the river barrier and do not show homeward orientation when released at this site (Pašukonis et al. 2014)
4. **Control (n = 7):** Frogs kept inside the holding barrel and sacrificed immediately after opening the container, at the same time of the day as the experimental group frogs. These frogs experience the same manipulations and circadian activity patterns, but are not exposed to a place recognition task.

Before releasing the frog, we determined the homeward orientation using the GPS points of the capture and release sites and a compass. We then set up a visual reference grid on the forest floor oriented towards the capture site and marked with four perpendicular 3-m strings. We released the frog at the center of the reference grid by gently tipping over the holding container and immediately retreated to at least three meters perpendicular to the homeward axis for observation. One observer continuously recorded each frog’s behavior for 120 min. The behavior of the first four frogs was recorded for 90 min (two frogs in home, one in familiar, and one in unfamiliar conditions), but the observation period was increased for all other individuals due to long periods spent immobile and hiding. We used the program JWatcher 1.0 (Blumstein et al. 2006) running on a portable computer (WinTab 9, Odys, Willich, Germany) to record the number of hops, turns, and feeding events and the time spent hiding under leaves, sitting on the ground, or perched on elevated structures at least 15 cm above the surrounding. Experimental frogs were never observed calling during trials. The tracking device was used to locate the frog if it disappeared out of sight, and the duration of the out-of-sight period was noted. At the end of the observation period, we measured the distance from the release site to the final frog location using a laser range-finder and the angular deviation from the release site relative to the capture site using a precision compass. The angular deviation represented the directionality of movement relative to the capture site, which was three meters away for the home release, 50 m away for the familiar release, and 200 to 600 m away for the unfamiliar release. Two frogs were excluded from behavioral data collection because they were lost during the observation, and another was excluded because the frog lost the tag before release. These three frogs, excluded from the brain sampling, were recaptured and released back at their home territories.

### Brain collection and processing

After observation, frogs in the home, unfamiliar, and familiar experimental groups were captured with a plastic bag, anesthetized with 20% benzocaine gel, weighed, measured, and euthanized by rapid decapitation. Seven control frogs were kept inside the holding containers and their brain samples were collected around the same time as the experimental group, immediately after opening the container, in a similar manner. All frogs were euthanized between 145 and 278 min after sunrise. To standardize the behavioral data, we did not collect the brains of frogs that hid for more than 60 min (n = 7) or moved more than 10 m from the release site (n = 3). The brain was typically dissected within 5 min after decapitation, but occasionally up to 10 min, and immediately fixed in 4% paraformaldehyde (PFA). A total of 30 brains were collected: 7 control, 8 home release, 8 familiar area release, and 7 unfamiliar area release. The brains were fixed in PFA overnight at 4 °C, rinsed in 1X phosphate buffered saline (PBS) and transferred to 30% sucrose in PBS with 0.1% sodium azide. Samples in sucrose were stored at 4 °C for up to one month until returning from the field. Brains were then embedded in mounting media (Tissue-Tek O.C.T. Compound, Electron Microscopy Sciences, Harfield, PA, USA), frozen on dry ice and stored at -80 °C until cryosectioning approximately 3 months later. We sectioned the whole brain into four coronal series at 14 µm. These sections were thaw-mounted onto SuperFrost Plus microscope slides (VWR International, Randor, PA, USA), allowed to dry completely, and then stored at -80 °C. One series was used for pS6 staining.

### Estimating neural activation

We used an antibody for phosphorylated ribosomes (pS6; phosphor-S6 Ser235/236; Cell Signaling, Danvers, MA, USA) to assess levels of neural activation in three pallial regions (medial (Mp), dorsal (Dp), and lateral pallium (Lp)), three subpallial regions (lateral septum (Ls), medial septum (Ms) and striatum (Str)) and the hypothalamic preoptic area (Poa). Ribosomes become phosphorylated when neurons are active and pS6 thus serves as a general marker of translationally active neurons, akin to immediate early genes (Knight et al. 2012). The peak detection of pS6 occurs from tens of minutes to 2 h depending on specific stimuli (Knight et al. 2012). We consider our experimental release a continuous stimulation, and thus, did not expect major differences between individuals euthanized after 90 min or 120 min of observation. We followed standard immunohistochemical procedures for 3’,3’-diaminobenzadine (DAB) antibody staining. Briefly, we quenched endogenous peroxidases using a 30% sodium hydroxide solution, blocked slides in 5% normal goat serum to reduce background staining, incubated slides in primary antibody (rabbit anti-pS6 at 1:500 in blocking solution) overnight, incubated slides in secondary antibody for 2 h, followed by avidin–biotin complex (Vectastain Elite ABC Kit; Vector Laboratories, Burlingame, CA, USA) solution incubation for two hours, and treatment with DAB (Vector Laboratories, Burlingame, CA, USA) for 2 min. We rinsed slides with 1X PBS before and after all the above steps. Finally, slides were rinsed in water, counterstained with cresyl violet, dehydrated in a series of ethanol baths (50%, 75%, 95%, 100%, 100%), and cleared with xylenes prior to cover slipping with permount.

Slides were imaged at 20X with Olympus VS120 slide scanner (Olympus Corportaion, Tokyo, Japan). Staining was visualized and quantified using the software QuPath version 0.2.2 (Bankhead et al. 2017). The six relevant regions were delineated based on morphological structures revealed by cresyl violet nuclear counterstain and region boundaries described in Westhoff and Roth (2002), Moreno and González (2004), and Dicke and Roth (2007) adapted for *A. femoralis.* We marked the boundaries of each region on each section, recorded the selected surface area, and manually counted all pS6-positive cells in the boundary. Cells were quantified in a single hemisphere for each region in each section where that brain region was entirely visible. Due to tissue damage related to storage and sectioning, the number of usable sections per individual per region varied from 2 to 21 (median = 10) for pallial areas, from 2 to 12 (median = 5) for the subpallial areas, and from 1 to 6 (median = 3) for the Poa.

### Statistical analyses

All statistical analyses were performed with R (version 4.4.2, R Development Core Team 2024) in RStudio (version 2024.09.0 + 375, Posit team 2025). Plots were generated with ggplot2 (version 4.0.0, Wickham 2016) and circular packages (version 0.5-1, Agostinelli and Lund 2024) in R. We first analyzed behavioral differences between experimental groups, including frogs that were excluded from brain sampling (n = 32). We fitted data-appropriate models for each behavioral data type followed by post-hoc pairwise comparisons between experimental conditions with least-squares means contrast and Tukey-correction for multiple comparison with ‘emmeans’ function within R package emmeans (version 2.0.1 Lenth and Piaskowski 2025). We evaluated model diagnostics using standardized residual plots. For the number of feeding attempts, we fitted a zero-inflated generalized linear model (GLM) with a negative binomial distribution using the ‘zeroinfl’ function with the package pscl 1.5.9 (version 1.5.9, Zeileis et al. 2008). For movement, we summed the number of body turns and hops into a total number of movements (i.e., moves). We then fitted a GLM with poisson error distribution using the ‘glm’ function within R package stats. To analyze the proportion of time spent hiding, on the ground, and perched on elevated structures we fitted three separate beta regression models for proportions via maximum likelihood using the ‘betareg’ function within the package betareg (version 3.2–4, Cribari-Neto and Zeileis 2010). To account for zero values in the proportions data, we used a data transformation following Verkuilen and Smithson (2012): x' = (x(N – 1) + s)/ N, where N is the samples size and s is a constant between 0 and 1 (0.5 in our case). To evaluate the directionality of frog movements during behavioral observation in relation to their capture site, we used the Rayleigh test of circular uniformity with a specified mean direction (i.e. each frog’s respective capture site) as an alternative hypothesis using the ‘rayleigh.test’ function within the R package circular. Frogs that moved less than 0.5 m from the release point during the observation period were excluded from this analysis. All the same analyses were repeated only with the individuals (n = 23) for which brains were collected. The results were overall qualitatively similar to the full-sample analyses (n = 32), but the sample size was too small for inferential statistics on the angular data. Therefore, we report the full-sample results for behavioral data and provide the subset results in the supplements.

For pS6 data, we fitted two different generalized linear mixed models (GLMM) with a negative binomial distribution using ‘glmmTMB’ function within the glmmTMB R package (version 1.1.14, Brooks et al. 2017). First, we fitted a GLMM with pS6-positive cell count as the response variable and experimental condition, brain region, and their interaction as fixed factors. Second, we fitted a GLMM with pS6-positive cell count as the response variable and experimental condition, brain region, interaction of condition and region, as fixed factors, and the number of movements and time spent on elevated structures as covariates. We chose to include these two behavioral variables as covariates because they showed significant differences between experimental conditions. We did not include three-way interactions among brain region, condition, and behavior because we were specifically interested in differences among brain regions while accounting for behavioral variables. For this second model, the control condition frogs were excluded since no behavioral observations were done for this group. For both models, frog identity was included as a random effect to account for repeated sampling of brain regions within individuals and the log-transformed brain region area was included as an offset to account for potential differences in brain size. We evaluated model diagnostics using residual plots obtained with package DHARMa (version 0.4.7, Hartig 2024). To evaluate and report the effects of each predictor, we calculated analysis-of-variance tables for each model using ‘Anova.glmmTMB’ within the glmmTMB package. To evaluate the differences in pS6-positive cell counts between experimental conditions, we followed up each GLMM with post-hoc pairwise comparisons between experimental conditions within each brain region with least-squares means contrast and Tukey-correction for multiple comparison with ‘emmeans’ function within emmeans R package. As the pS6-positive cell count in the Ls and Ms were strongly correlated and results identical in all models, we merged the two regions into a single septum (Sep) for all analyses and plotting. The results for separate Ls and Ms are provided in the supplements.

## Results

### Frog behavior changes based on release site

**Fig. 1:**
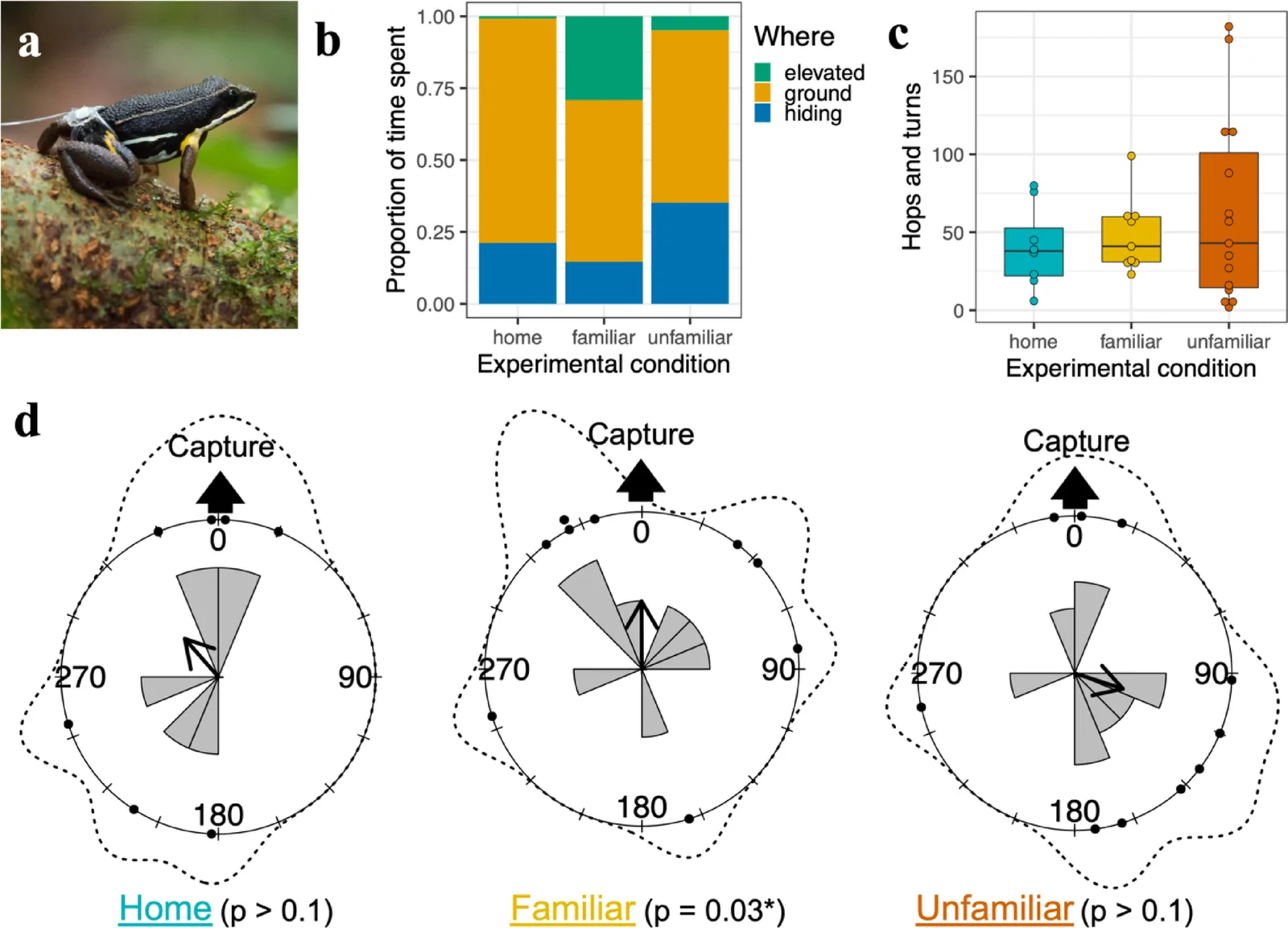
Frog behavior varies by release site. **a** Photograph of a male *A. femoralis* perched on an elevated structure wearing a tracking tag. **b** Proportions of time spent perched on elevated structures, on the ground, and hiding. Frogs released in a familiar area spent significantly more time perched on structures. **c** Counts of hops and turns (total movement) showing the most movement and the biggest variation in frogs released in an unfamiliar site. **d** Directionality of initial movements in relation to the capture site for each experimental condition. Only frogs released at a familiar site away from home showed significant orientation towards the capture sites

Frog behavior was observed immediately after release, including movement, location, and feeding (Fig. 1). Frogs released at a familiar area away from home spent more time perched on elevated structures than frogs released at home (lsm contrast home-familiar: *β* = -2.3, *p* = 0.01) or in an unfamiliar area (lsm contrast familiar–unfamiliar: *β* = 2.1, *p* = 0.005, Fig. 1b). Frogs moved the least when released at the home territory (lsm contrast home-familiar: *β* = -0.17, *p* = 0.05) and most when released in an unfamiliar area (lsm contrast familiar-unfamiliar: *β* = -0.26, *p* < 0.001, Fig. 1c.) Only the frogs released in a familiar area showed significant orientation towards the capture site (Rayleigh *p* = 0.03, CI low = 87.7°, CI high = -61.5°, reference angle = 0°, Fig. 1d). There were no differences between experimental groups in the number of feeding attempts, proportion of time spent hiding or proportion of time spent on the ground.

### Neural activity changes based on the release site

**Fig. 2:**
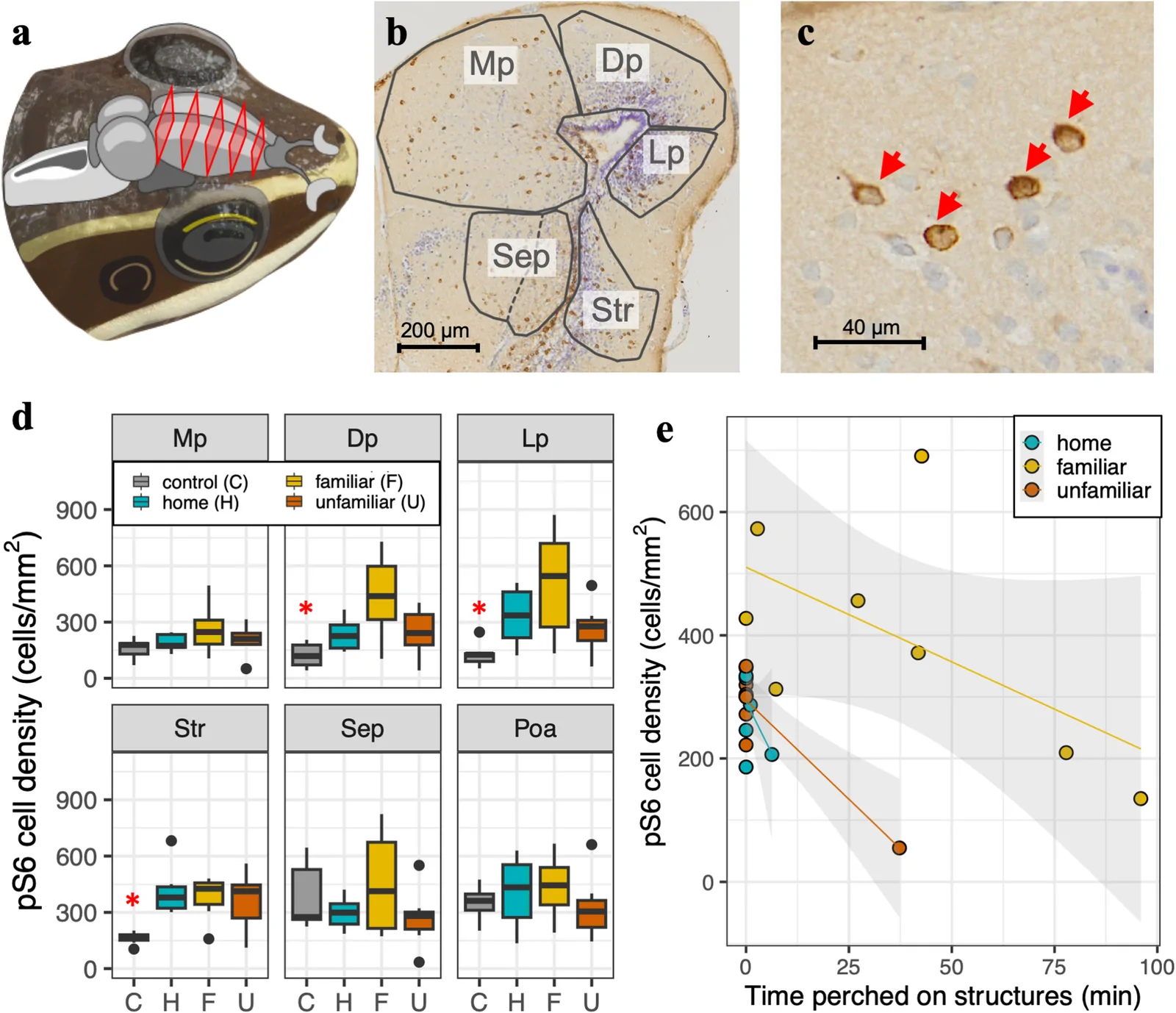
Neural correlates of spatial orientation in *Allobates femoralis* measured by translationally active cells. **a** Schematic representation of coronal telencephalic sections (in red) used to evaluate brain translational activity. **b** Example of an annotated coronal section through mid-telencephalon stained for immunopositive ps6-positive cells and counterstained with cresyl violet for cell nuclei. Grey outlines show the delineation of telencephalic brain regions where ps6-positive cells were counted: medial pallium (Mp), dorsal pallium (Dp), lateral pallium (Lp), striatum (Str), and septum (Sep). The dotted line indicated the separation between the lateral and medial septa. **c** Close-up of medial pallium with red arrows indicating four examples of ps6-positive stained cells. **d** Differences in the density of ps6-positive cells per region for each experimental condition: *C* control in grey, *H* home in blue, *F* familiar in yellow, *U* unfamiliar in red. Plotted values represent the pS6-positive cell density, averaged per region and per individual. Boxplot rectangles indicate the lower and upper quartiles with the median line, and whiskers extend to 1.5 times the interquartile range. Red asterisks indicate where the control condition was significantly different from experimental conditions based on the least-squares means contrast in Table 2. **e** Association between time perched on elevated structures and the total ps6-positive cell density across all evaluated regions: Mp, Lp, Lp, Str, Ls, and Poa, color-coded by experimental condition. Each dot represents the total pS6-positive cell density averaged per individual

Given the behavioral differences of frogs in different release conditions, we next asked if there were changes in the number of neurons that are translationally active (i.e. pS6-positive cells) across several brain regions important in social and spatial behavior (Fig. 2). We found a significant interaction between the experimental release condition and brain region (GLMM interaction: *X*^2^ = 182.3, df = 15, *p* < 0.001; Table 1). Pair-wise comparisons within each brain region showed the control condition had fewer pS6-positive cells than all other conditions in the lateral pallium (Lp), dorsal pallium (Dp), and striatum (Str), but not in medial pallium (Mp), septum (Sep), or preoptic area (Poa) (Table 2, Fig. 2e). No significant pair-wise comparison between experimental conditions was found in post-hoc testing, although the familiar condition tended to have more ps6-positive cells in the Dp and Lp (Fig. 2d).

**Table 1:** Anova table of the generalized linear mixed model testing the effects of experimental condition (condition), brain region (region), and their interaction on ps6-positive cell count. Individual included as a random factor

Predictor	${\chi }^{2}$	df	*p*
**Region**	194.3	5	** < 0.001**
**Condition**	26.1	3	** < 0.001**
Region*Condition	182.3	15	** < 0.001**

${\chi }^{2}$ test statistic, *df* degrees of freedom, *p* probability value

Bold values indicate *p* < 0.05

**Table 2:** Least-squares means contrast of experimental conditions for each brain region based on the GLMM shown in Table 1

	Medial pallium				Dorsal pallium			
Contrast	Est	SE	z.ratio	*p*	Est	SE	z.ratio	*p*
**Control—Home**	-0.24	0.23	-1.05	0.72	-0.66	0.23	-2.86	**0.02**
**Control—Familiar**	-0.43	0.23	-1.87	0.24	-1.19	0.23	-5.10	** < 0.001**
**Control—Unfamiliar**	-0.17	0.24	-0.73	0.89	-0.63	0.24	-2.64	**0.04**
Home—Familiar	-0.19	0.22	-0.86	0.83	-0.52	0.22	-2.36	0.08
Home—Unfamiliar	0.07	0.23	0.30	0.99	0.03	0.23	0.13	1.00
Familiar—Unfamiliar	0.26	0.23	1.13	0.67	0.55	0.23	2.41	0.07

	Lateral pallium				Striatum			
Contrast	Est	SE	z.ratio	*p*	Est	SE	z.ratio	*p*
**Control—Home**	-0.99	0.26	-3.86	** < 0.001**	-0.92	0.25	-3.74	** < 0.001**
**Control—Familiar**	-1.25	0.26	-4.85	** < 0.001**	-0.95	0.25	-3.82	** < 0.001**
**Control—Unfamiliar**	-0.77	0.27	-2.91	**0.02**	-0.74	0.25	-2.90	**0.02**
Home—Familiar	-0.26	0.23	-1.12	0.68	-0.02	0.23	-0.10	1.00
Home—Unfamiliar	0.22	0.24	0.92	0.79	0.19	0.24	0.78	0.86
Familiar—Unfamiliar	0.48	0.24	2.00	0.19	0.21	0.24	0.88	0.82

	Septum				Preoptic area			
Contrast	Est	SE	z.ratio	*p*	Est	SE	z.ratio	*p*
Control—Home	0.25	0.26	0.97	0.76	-0.32	0.25	-1.28	0.58
Control—Familiar	-0.06	0.26	-0.22	1.00	-0.26	0.25	-1.04	0.73
Control—Unfamiliar	0.48	0.27	1.79	0.28	0.30	0.26	1.14	0.67
Home—Familiar	-0.31	0.25	-1.23	0.61	0.06	0.24	0.27	0.99
Home—Unfamiliar	0.23	0.26	0.88	0.82	0.62	0.26	2.40	0.08
Familiar—Unfamiliar	0.53	0.26	2.07	0.16	0.56	0.25	2.20	0.12

*Est* estimate, *SE* standard error, *z.ratio* test statistic, *p* probability value

Bold values indicate *p* < 0.05

As significant differences were found in the number of movements and time perched on elevated structures between experimental conditions (Fig. 1), we next examined whether pS6-positive cell numbers differed between conditions when accounting for behavioral differences (Table 3). We found again a significant interaction between experimental condition and brain region (GLMM interaction: *X*^2^ = 51.9, df = 10, *p* < 0.001) and a significant negative effect of time elevated on structures (GLMM time elevated, *X*^2^ = 0.3, df = 1, *p* < 0.001), but not of the number of movements (Table 3). Visual inspection of correlation plots (Fig. 2e) suggested that the effect of time spent on elevated structures might be driven by two individuals released in familiar area and with the longest time spent on elevated structures. To evaluate the influence of these two individuals, we repeated the model excluding them and found a similar overall trend (GLMM time elevated: *X*^2^ = 3.6, df = 1, *p* = 0.06). Therefore, we report the results of the full model, including all individuals. Due to this relationship with behavior, pairwise comparisons revealed significant differences between experimental conditions in all brain regions (Table 3, 4). Specifically, frogs released in a familiar area had significantly more pS6-positive cells in Dp, Lp, Mp, Sep, and Str than frogs released back home or in an unfamiliar area (Table 4). In addition, frogs released in an unfamiliar area had significantly fewer pS6-positive cells in Poa than frogs released back home or in familiar area (Table 4).

**Table 3:** Anova table of the generalized linear mixed model testing the effects of experimental condition (condition), brain region (region), number of movements (moves), and time spent on elevated structures (time elevated) on ps6-positive cell count. Individual included as a random factor

Predictor	${\chi }^{2}$	df	*p*
**Region**	194.5	5	** < 0.001**
**Condition**	31.3	2	** < 0.001**
Moves	20.3	1	0.61
**Time elevated**	0.3	1	** < 0.001**
Region*Condition	51.9	10	** < 0.001**

${\chi }^{2}$ test statistic, *df* degrees of freedom*, p* probability value

Bold values indicate *p* < 0.05

**Table 4:** Least-squares means contrast of experimental conditions for each brain region based on the GLMM shown in Table 3

	Medial pallium				Dorsal pallium			
Contrast	Est	SE	z.ratio	*p*	Est	SE	z.ratio	*p*
**Home—Familiar**	-0.74	0.21	-3.56	**0.001**	-1.08	0.21	-5.11	** < 0.001**
Home—Unfamiliar	0.00	0.18	-0.03	1.00	-0.04	0.18	-0.24	0.97
**Familiar—Unfamiliar**	0.74	0.20	3.61	** < 0.001**	1.04	0.21	5.01	** < 0.001**

	Lateral pallium				Striatum			
Contrast	Est	SE	z.ratio	*p*	Est	SE	z.ratio	*p*
**Home—Familiar**	-0.82	0.22	-3.65	** < 0.001**	-0.58	0.22	-2.60	**0.03**
Home—Unfamiliar	0.15	0.19	0.78	0.71	0.11	0.19	0.60	0.82
**Familiar—Unfamiliar**	0.97	0.22	4.39	** < 0.001**	0.69	0.22	3.17	** < 0.001**

	Septum				Preoptic area			
Contrast	Est	SE	z.ratio	*p*	Est	SE	z.ratio	*p*
**Home—Familiar**	-0.86	0.24	-3.60	** < 0.001**	-0.49	0.23	-2.11	0.09
**Home—Unfamiliar**	0.15	0.21	0.72	0.75	0.55	0.21	2.57	**0.03**
**Familiar—Unfamiliar**	1.01	0.24	4.27	** < 0.001**	1.03	0.23	4.45	** < 0.001**

*Est* estimate, *SE* standard error, *z.ratio* test statistic, *p* probability value

Bold values indicate *p* < 0.05

## Discussion

In this study, we investigated the behavior and neural correlates of frogs after displacement and release at locations presenting different challenges for spatial orientation. Frogs released at distant and familiar sites showed distinct behavioral responses that were partially reflected in differential neural activity patterns. We discuss our results in relation to our a priori predictions and the existing literature on the neural basis of navigation in amphibians.

Behavioral responses observed within the first two hours after release indicate that frogs discriminate between their home territory, familiar environments surrounding their territory, and unfamiliar environments. We specifically targeted this initial orientation phase before active homing because we presume that the sensory-cognitive integration necessary for navigation occurs during this period, as supported by our behavioral observations. As in previous studies on *A. femoralis* (Pašukonis et al. 2014, 2022), only frogs released in a familiar environment, but outside their home territories, showed a significant orientation towards the capture site. Most frogs in this group stayed within a few meters of the release point, often immobile or moving between, and occasionally perched on, elevated structures. Similar observations were reported in previous studies on *A. femoralis* (Pašukonis et al. 2014) and related species (Pašukonis et al. 2018), where this latent phase was followed by a direct map-like orientation and movement toward the home territory. Frogs released back into their home territory do not exhibit this orientation behavior, as they move little and do not use elevated structures, suggesting that they immediately recognize it. In contrast, frogs released at unfamiliar sites on average moved the most, which we interpret as exploratory behavior of unfamiliar areas. Interestingly, five of the seven frogs excluded from brain sampling because they hid during the observation period were in an unfamiliar environment, and all three frogs that moved out of our observation area were from the group released in an unfamiliar area. This divergent “run or hide” response suggests that frogs immediately react to an unfamiliar environment as a stressor.

As in other vertebrates, the medial pallium (Mp) in amphibians has been associated with spatial behavior in mazes (Sotelo et al. 2017, 2022, 2024) and homing behavior in the field (Shaykevich et al. 2025). Although we initially predicted that the Mp would show more pS6-positive cells in frogs released at familiar sites, we did not detect a strong selective pattern in the Mp during the navigational task. In the first analysis, we found that Mp activation in the control group, in which frogs remained in a dark container after capture, did not differ from that in the experimental conditions. All frogs were kept in containers overnight, and while visual and olfactory cues were minimized, all frogs were in the same acoustic environment. Sotelo et al. (2020) have also shown that Mp is involved in a spatial learning task using a conspecific call as a cue. Therefore, it is possible that our control group was acoustically exposed to a type of place recognition task similar to that of the frogs released in an unfamiliar area. It is likely that the complexity of the natural environment, combined with our relatively small sample sizes, masks more context-specific signal in the Mp. Along those lines, a more detailed analysis accounting for behavior among experimental conditions revealed higher Mp activation in frogs orienting home than in frogs released back home or in an unfamiliar area. We speculate that frogs orient in space while perched on elevated structures, thereby reducing background noise from overall brain activity related to other behaviors (e.g., movement, feeding) and revealing a navigation-specific pattern of brain activation.

Amphibian Mp is the largest pallial area with a relatively simple neuroanatomical architecture, likely involved in a variety of integration tasks (Striedter and Northcutt 2019; Sotelo et al. 2024). Several studies have shown that poison frog navigation relies on spatial memory (Stynoski 2009; Pašukonis et al. 2016; Ringler et al. 2016) and some familiarity with local cues (Pašukonis et al. 2014). A recent field study on homing in the cane toad *R. marina* found a strong selective activation of Mp in individuals that were approaching home after displacement, in contrast to individuals that were not displaced or unable to home (Shaykevich et al. 2025). In contrast, we collected the brain tissue at the initial phase of orientation, therefore measuring initial orientation and information integration rather than homing behavior. This key difference in the time point of brain tissue collection might strongly influence the neuronal response pattern. In support of this idea, hippocampal lesions in homing pigeons selectively affect the final stage of homing when landmarks are used, but not the initial orientation that relies on large-scale olfactory gradients and sun-compass (Bingman et al. 1984; Gagliardo et al. 2009). In contrast to lab studies on amphibians or field studies on homing pigeons, we do not know which sensory cues or cognitive mechanisms frogs use to navigate from a familiar area after translocation, which limits our interpretations of the observed neural activation patterns. While different sub-regions and cellular types have been identified in the amphibian pallium (Roth and Westhoff 1999; Woych et al. 2022), their functional significance in different spatial and non-spatial tasks remains unknown.

In mammals, initial exploration of an unfamiliar environment relies on path integration and route learning associated with subpallial structures such as the striatum (Str) and the septum (Sep) (Packard and McGaugh 1996; McNaughton et al. 2006; Chersi and Burgess 2015; Hinman et al. 2018). These regions did not show more pS6-positive cells when frogs were exploring an unfamiliar area. Instead, the initial analysis revealed that Dp, Lp, and Str showed more pS6-positive cells in frogs expected to home from a familiar area in comparison to the control group (i.e., no release exposure). The more detailed analyses between experimental groups revealed that all measured regions (Mp, Dp, Lp, Str, Sep), except for the Poa, showed increased activation in frogs released in a familiar area outside of their home territory when compared to frogs released back home or at an unfamiliar site. Therefore, we speculate that these regions are involved in sensory-cognitive integration required for homing in a familiar area, whereas frogs in an unfamiliar area are not able to navigate, and frogs back home do not need to navigate.

Our findings support the view that Dp and Lp play a role in navigating when familiar cues are available, including in large-scale natural environments. The developmental and functional homologies and the exact boundaries of the dorsal and lateral pallia in amphibians are still being debated. Traditionally, these regions have been considered homologous to the mammalian general and piriform cortices, respectively (Bruce and Neary 1995; González et al. 2020). However, recent studies suggest the amphibian Dp has similarities with the entorhinal cortex and subiculum (Woych et al. 2022), which play a key role in spatial cognition. Other authors have also drawn potential homologies between Lp and mammalian piriform and entorhinal cortices (Laberge and Roth 2007; Roth et al. 2007). On the functional level, authors have broadly described the Dp and Lp as integrative-associative limbic areas (Westhoff and Roth 2002; Roth et al. 2007). Studies by Sotelo et al. (2017), Wenz and Himstedt (1990), and Sotelo et al. (2024) suggest that Dp and Lp might be specifically involved in visual cue integration for spatial orientation. However, it is important to note that spatial tasks in the lab typically rely on visual cues, while amphibians navigating in the wild also rely on olfactory, acoustic, and magnetic information (Ferguson 1971; Sinsch 1990). For example, a recent study found an increased Lp activation in toads navigating from long distances in the field (Shaykevich et al. 2025). Furthermore, unlike in mammals, amphibian Lp projects directly to and receives input from the olfactory bulb (Roth et al. 2007). Therefore, Lp and Dp might be implicated in integrating a variety of sensory cues required to navigate in the field.

Increased activation observed in other regions, such as Str, Sep, and Poa, was also correlated with release in familiar environments were frogs in unfamiliar areas had fewer pS6-positive cells than those in familiar areas or home territories. In addition, the Poa showed the highest number of pS6-positive cells when the male was released back into his home territory, especially compared to an unfamiliar area. We expected increased Str and Sep activity in unfamiliar areas, because in mammals, these regions are associated with path integration and the use of egocentric cues that typically predominate when exploring an unfamiliar environment. It remains unclear to what extent amphibians exploring an unfamiliar area rely on path integration or route learning, so the connection or lack thereof between the observed activation pattern and path integration remains speculative. However, the increased activity of Str and Sep in frogs orienting from familiar places after translocation suggests that these regions may be also implicated in navigation using mechanisms other than route- and cue-based learning or path integration. Increased Poa activity in males released back home fits the general patterns of Poa modulating social behaviors, such as territoriality and parental care across vertebrates (Demski and Knigge 1971; Numan and Insel 2003; Ruscio and Adkins-Regan 2004). Poa has also been shown to modulate parental care in poison frogs (Fischer et al. 2019; Fischer and O’Connell 2020), and territorial males used in this study are also the primary care-giving sex in *A. femoralis* (Ringler et al. 2015).

## Summary

Overall, we find that wild poison frogs respond selectively to the familiarity of the environment and that most measured pallial and sub-pallial areas are more activated when frogs are challenged to orient in a familiar environment. Contrary to our predictions, the specific activation patterns do not reveal a selective role of the medial pallium in this navigation task, and responses are more prominent in the dorsal and lateral pallia. Overall, our results suggest that the activity in the medial, dorsal, and lateral pallia, along with the striatum and septum, is associated with amphibian spatial behavior in the wild. Our interpretations here are limited by the sparsity of studies on amphibian spatial cognition, the noise and limited sample sizes of field studies on wild vertebrates, and unresolved homologies of vertebrate brain regions concerned. However, this study reinforces the idea that amphibians are a tractable system for neuroethological studies in the wild, where consistent behavioral and neural responses can be elicited by experimental manipulations. Our findings provide important inroads into understanding the neural and sensory basis of amphibian spatial cognition, especially in natural environments.

## Supplementary Information

Below is the link to the electronic supplementary material.

Supplementary Material 1

Supplementary Material 2

Supplementary Material 3

Supplementary Material 4

Supplementary Material 5

Supplementary Material 6

Supplementary Material 7

Supplementary Material 8

Supplementary Material 9

## Data Availability

All data and scripts are provided with the supplementary materials.
