# Correction: Neural correlates of spatial orientation in a territorial frog *Allobates femoralis*

**DOI:** 10.1007/s00359-026-01849-7

**Published:** 2026-09-25

**Authors:** Andrius Pašukonis, Eva K. Fischer, Lauren A. O‘Connell

**Affiliations:** 1 Institute of Biosciences, Life Sciences Center, Vilnius University, Vilnius, Lithuania; 2 Department of Interdisciplinary Life Sciences, Konrad Lorenz Institute of Ethology, University of Veterinary Medicine Vienna, Vienna, Austria; 3 Department of Neurobiology, Physiology and Behavior, University of California Davis, Davis, CA USA; 4 Department of Biology, Stanford University, Stanford, CA USA; 5 Wu Tsai Institute for Neuroscience, Stanford University, Stanford, CA USA

**Correction: Journal of Comparative Physiology A**

**https://doi.org/10.1007/s00359-026-01820-6**

After publication of this article, the authors discovered that in Fig. 2d the red asterisk was erroneously placed so that a significant difference was indicated in the Medial pallium (Mp) rather than in the Dorsal pallium (Dp).

**Figure:**
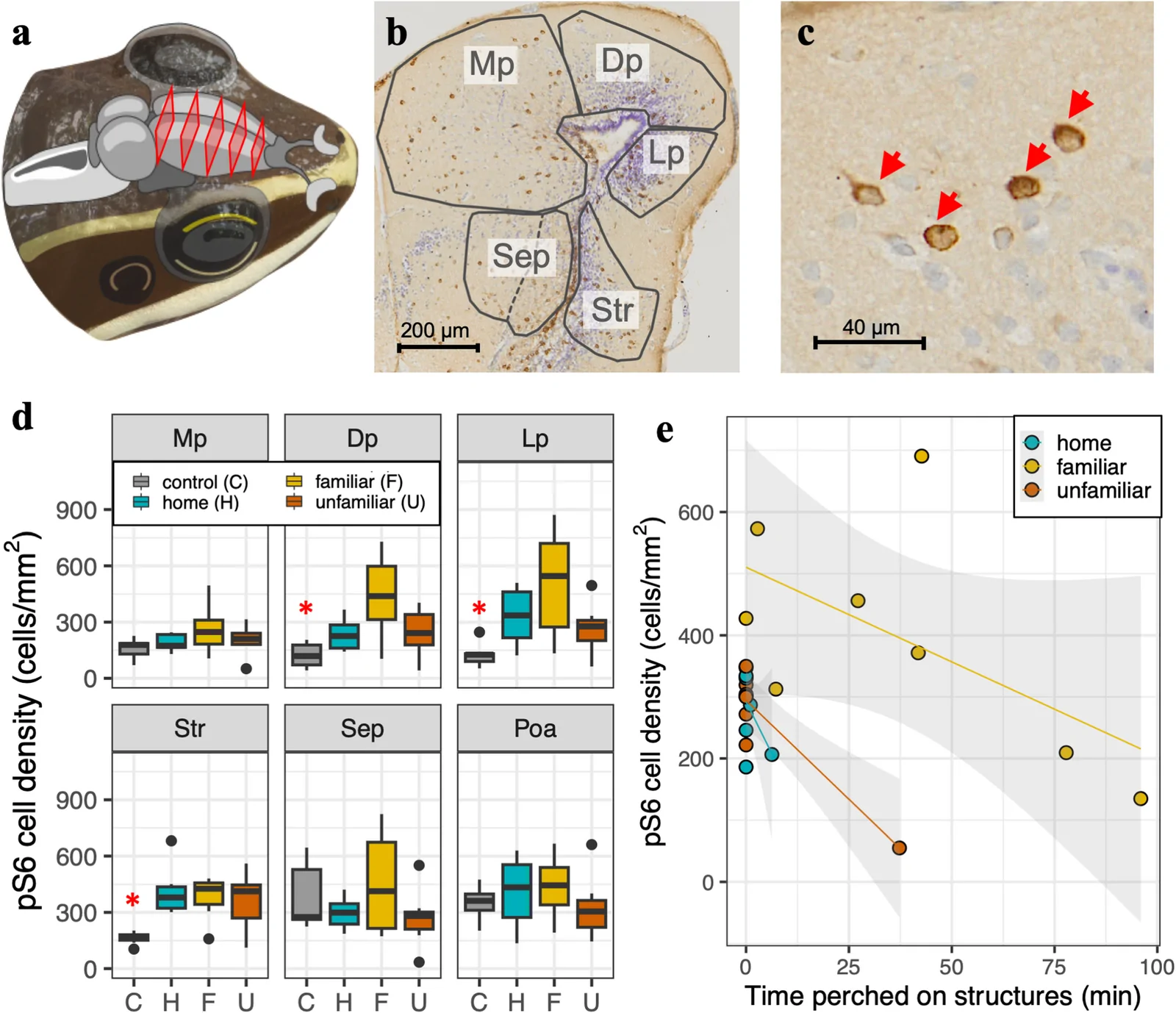

The error in the original article has been corrected.

